# SUMOylation disassembles the tetrameric pyruvate kinase M2 to block myeloid differentiation of leukemia cells

**DOI:** 10.1038/s41419-021-03400-9

**Published:** 2021-01-20

**Authors:** Li Xia, Yue Jiang, Xue-Hong Zhang, Xin-Ran Wang, Ran Wei, Kang Qin, Ying Lu

**Affiliations:** 1 Institute of Dermatology, Xinhua Hospital, Key Laboratory of Cell Differentiation and Apoptosis of the Chinese Ministry of Education and Department of Core Facility of Basic Medical Sciences, Shanghai Jiao Tong University School of Medicine, Shanghai, China; 2 Department of Hematology, Dalian Key Laboratory of Hematology, Liaoning Medical Center for Hematopoietic Stem Cell Transplantation, The Second Hospital of Dalian Medical University, Dalian, China

**Keywords:** Leukaemia, Haematological cancer

## Abstract

Leukemia arises from blockage of the differentiation/maturation of hematopoietic progenitor cells at different stages with uncontrolled proliferation of leukemic cells. However, the signal pathways that block cell differentiation remain unclear. Herein we found that SUMOylation of the M2 isoform of pyruvate kinase (PKM2), a rate-limiting glycolytic enzyme catalyzing the dephosphorylation of phosphoenolpyruvate to pyruvate, is prevalent in a variety of leukemic cell lines as well as primary samples from patients with leukemia through multiple-reaction monitoring based targeted mass spectrometry analysis. SUMOylation of PKM2 lysine 270 (K270) triggered conformation change from tetrameric to dimeric of PKM2, reduced PK activity, and led to nuclear translocation of PKM2. SUMO1 modification of PKM2 recruits and promotes degradation of RUNX1 via a SUMO-interacting motif, resulting in blockage of myeloid differentiation of NB4 and U937 leukemia cells. Replacement of wild type PKM2 with a SUMOylation-deficient mutant (K270R) abrogated the interaction with RUNX1, and the blockage of myeloid differentiation in vitro and in xenograft model. Our results establish PKM2 as an essential modulator of leukemia cell differentiation and a potential therapeutic target, which may offer synergistic effect with differentiation therapy in the treatment of leukemia.

## Introduction

The pyruvate kinase (PK) is a rate-limiting glycolytic enzyme catalyzing the dephosphorylation of phosphoenolpyruvate to pyruvate, yielding one molecule of ATP. The PK consists of four isoforms—the *PKLR* gene-encoded PKL and PKR isoforms and the *PKM* gene-encoded M1 (PKM1) and M2 (PKM2) isoforms. PKM1 is expressed in normal adult tissues that require high levels of energy, such as the heart, brain, and skeletal muscle and constitutively forms stable tetramers (the active form of PK). In contrast, PKM2, the dominant form of PK in tumors, exists in either tetramers or dimers with less activity which is allosterically regulated by fructose-1,6-bisphosphate (FBP)^1,2^. Oncogenic role of PKM2 was constructed based upon a large number of reports showing that aberrant expression of PKM2 promotes tumor cell growth through both metabolic and non-metabolic mechanisms^3–9^. Our previous works also demonstrated that PKM2 endows tumor cells with growth advantage, and genomic instability through interacting with P53 and functioning as a protein kinase of histone H2AX^10–12^. Notably, multiple post-translational modifications including acylation, phosphorylation, methylation, oxidation, and O-GlcNAcylation of PKM2 have been identified to powerfully modulate its activity^13–21^, which occur in response to various stimuli during tumor initiation or maintenance^13,17,20,22^. However, controversial evidences also demonstrated that PKM2 is dispensable for tumor development, suggesting a tissue specific function of PKM2 and obscuring its role as a therapeutic target^23–26^.

SUMOylation is a reversible covalent attachment of a small ubiquitin-related modifier (SUMO) protein to a target protein via the concerted action of the E1-activating enzyme, E2-conjugating enzyme, and E3-ligases^27–29^. This modification controls protein conformation, stability, interaction, and subcellular localization and therefore has been implicated in multiple pivotal cellular processes including cell cycle, apoptosis, metabolism, and stress adaptation^30–32^. Dysfunction of the SUMOylation enzymes may lead to severe defects in cell proliferation and genome stability and is involved in multiple types of cancer^33^. Moreover, inhibition of SUMOylation preferentially induces death of Myc-overexpressing solid tumors and restores apoptosis of chemo-resistant leukemia cells, suggesting SUMO pathway as an anticancer target^34–36^. Despite the intensive investigation, the contribution of SUMOylation in cancer development, particularly in hematopoietic malignancy is largely unclear. Herein, we reported that SUMOylation of PKM2 is prevalent in primary acute myeloid leukemia patient samples and cultured cell lines. SUMO1 modification of PKM2 at lysine 270 increased its nuclear localization and dimeric formation. Expression of PKM2 inhibits myeloid differentiation of leukemia cells, which could be abrogated by substitution of lysine 270 of PKM2 with arginine. Finally, we showed that SUMO1 modification of PKM2 at K270 is essential in recruiting SUMO-interacting motif (SIM)-bearing Runt-related transcription factor 1(RUNX1), a master transcriptional factor implicated in the differentiation of leukemia cells, providing a molecular basis for PKM2 as a potential target which may offer synergistic effect with differentiation therapy in the treatment of leukemia.

## Results

### SUMOylation of PKM2 at K270 is prevalent in leukemia cells

**Fig. 1:**
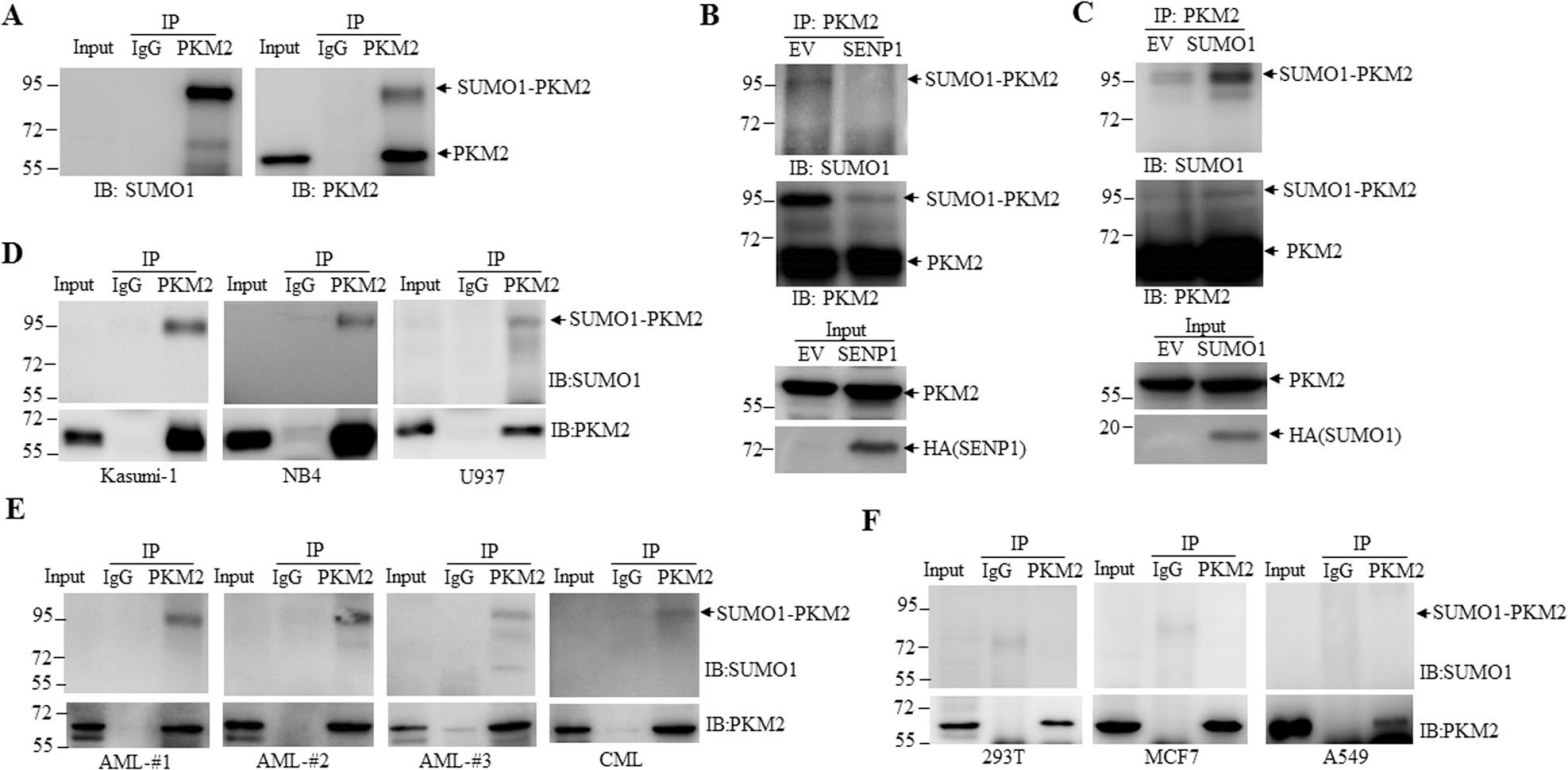
SUMOylation of PKM2 is prevalent in leukemia cells. **A** NB4 cells were transfected with Flag-tagged PKM2 and IP were performed with anti-PKM2 antibody followed by western blot with anti-SUMO1 and anti-PKM2 antibodies. **B**, **C** NB4 cells were transfected with plasmids encompassing SENP1 (**B**) or SUMO1 (**C**) and SUMOylation of PKM2 were analyzed. EV empty vector. **D**–**F** Endogenous SUMO1 modification of PKM2 in leukemia cells lines (**D**), primary mononuclear cells from bone marrow of patients with acute myeloid leukemia (AML) or chronic myeloid leukemia (CML) (**E**) and adherent cell lines (**F**) were measured via IP with PKM2 antibody followed by western blot with anti-SUMO1 or anti-PKM2 antibody.

To investigate whether PKM2 is SUMOylated in leukemia cells, we transiently transfected Flag-PKM2 into NB4 leukemia cells and performed immunoprecipitation (IP) with anti-Flag antibody. The result showed that anti-Flag antibody greatly enriched a band with a size of Mr ~95 kDa (the expected normal size of PKM2 is 58 kDa), which could also be strongly detected by anti-SUMO1 antibody (Fig. 1A). Moreover, we observed decrease and increase of SUMOylation of exogenous PKM2 by ectopically expressing sentrin/SUMO-specific protease 1 (SENP1) (Fig. 1B), or SUMO1 (Fig. 1C) in NB4 leukemia cell lines respectively, suggesting that PKM2 is a SUMOylated protein in leukemia cells. Importantly, endogenous SUMOylated-PKM2 could be detected in a number of leukemia cell lines (Fig. 1D) as well as primary bone marrow mononuclear cells (BMMC) samples from acute myeloid leukemia (AML) or chronic myeloid leukemia (CML) patients (Fig. 1E) but not adherent cell lines (Fig. 1F).

**Fig. 2:**
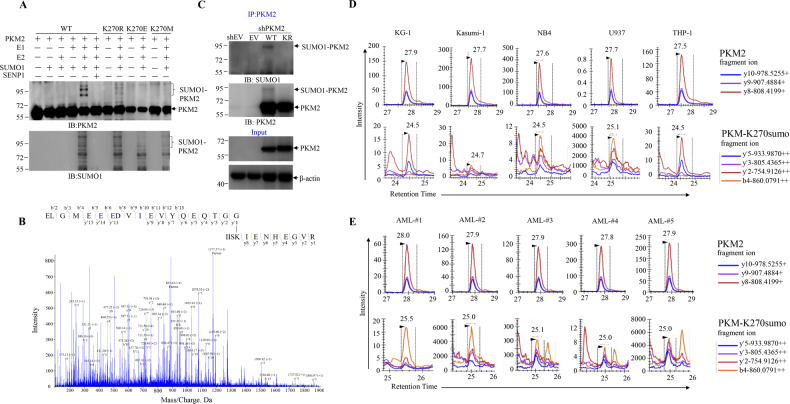
K270 is the major SUMOylation site in PKM2. **A** In vitro SUMOylation of PKM2. Recombinant wild type (WT) or K270R mutant His-tagged PKM2 were incubated with recombinant SUMO1 in the presence of purified E1(SAE1/UBA2) and E2(UBC9) with or without SENP1. SUMO1 modification were detected using antibody against PKM2 or SUMO1. K, lysine; R, arinine; E, glutamic acid; M, methionine. **B** SUMOylated PKM2 peptide identified by MS. Recombinant PKM2 protein was digested by trypsin and subjected to LC-MS/MS for identification. The lysine conjugated with QTGG remnant indicates the SUMOylation site. **C** WT or K270R mutant (KR) Flag-tagged PKM2 were infected into NB4 cells that endogenous PKM2 were depleted. The SUMO1 modification were measured by IP with anti-PKM2 antibody. **D**, **E** Multiple-reaction monitoring mass spectrometry(MRM-MS) analysis of SUMOylated PKM2 in leukemia cell lines (**D**) and primary samples from patients with AML (**E**).

To identify the possible lysine(K) residues of PKM2 that may be conjugated by SUMO1, we set up in vitro SUMOylation system showing that recombinant PKM2 could be directly SUMOylated by E1(SAE1/UBA2) combined with E2(UBC9) (Fig. 2A). Subsequent mass spectrometry (MS) technology identified a SUMO1 peptide covalently conjugated at K270 of PKM2 (Fig. 2B). The SUMOylation at K270 of PKM2 was further validated using in vitro SUMOylation system showing that mutation of K270 abrogated the SUMO1 conjugation of recombinant PKM2 (Fig. 2A). Moreover, transfection of K270R mutant PKM2 (KR) into NB4 cells displayed significantly lower level of SUMOylation compared to its wild type(WT) counterpart (Fig. 2C).

A major challenge of detecting post-translational modification is lack of site-specific and modification-specific antibodies. Moreover, low throughput of antibody-based measurement restrains it from application. Therefore, taken the SUMO1-conjugated PKM2 peptide identified from in vitro SUMOylation reaction as a target, we applied multiple reaction monitoring-based targeted MS (MRM-MS) to verify endogenous SUMOylation of PKM2 at K270 in leukemia cells. The results showed that although varying in abundance, SUMO1-conjugated K270-containing peptide of PKM2 was observed across a number of leukemia cell lines (Fig. 2D) and BMMC from leukemia patients (Fig. 2E), clearly demonstrating that endogenous SUMOylation of PKM2 is prevalent in leukemia cells.

### SUMOylation of PKM2 destabilizes PKM2 tetramer, reduces PK activity and promotes PKM2 nuclear localization

**Fig. 3:**
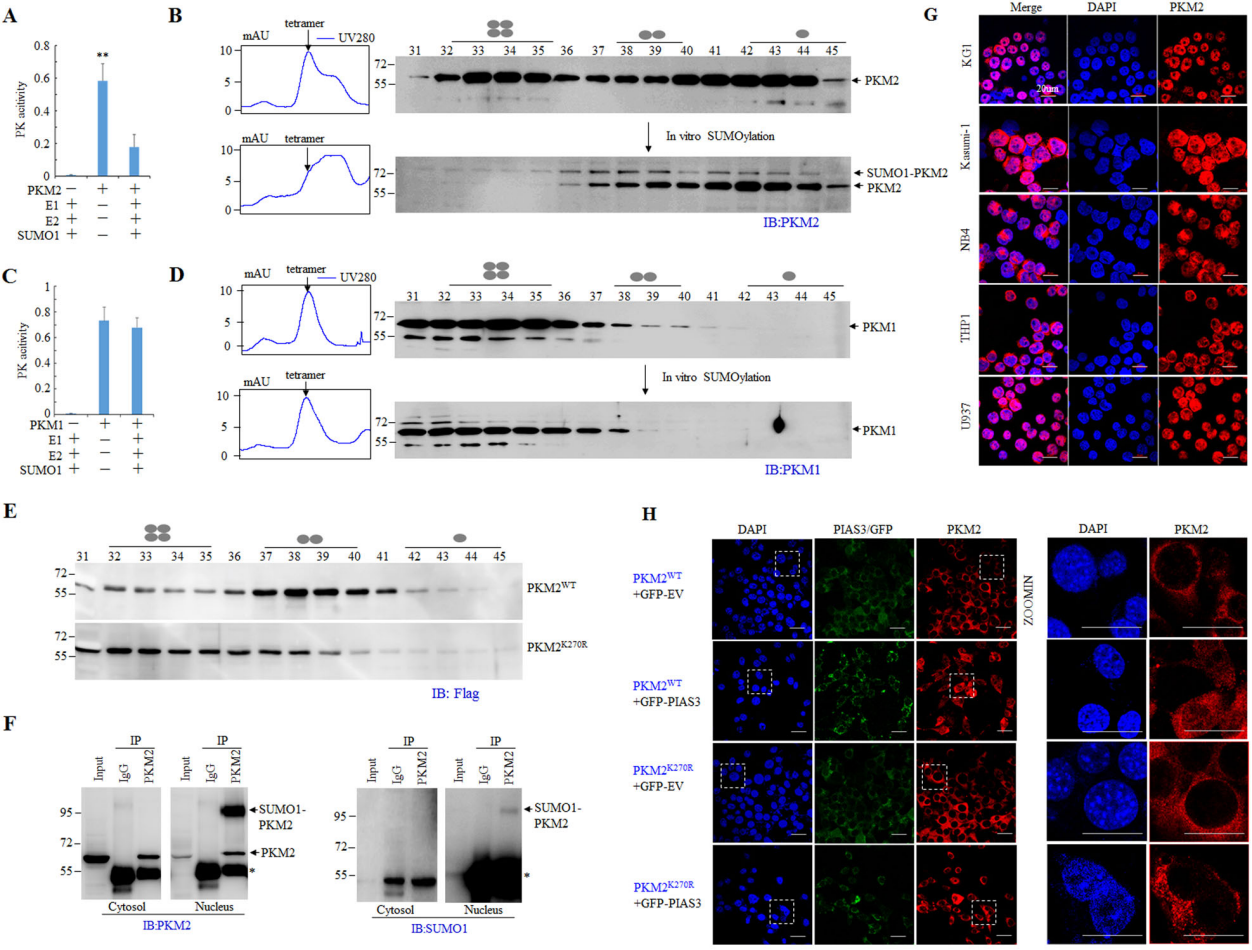
SUMO1 modification suppresses pyruvate kinase of PKM2, disassembles PKM2 tetramer and induces nuclear localization of PKM2. **A**, **C** In vitro SUMOylation reaction were performed by incubating recombinant PKM2 (**A**) or PKM1 (**C**) with purified SUMO1, E1 and E2. The samples were subjected to pyruvate kinase assay. **B**, **D** Size-exclusion chromatography analysis of samples from in vitro SUMOylation reaction followed by western blot analysis with anti-PKM2 (**B**) or anti-PKM1 (**D**) antibody. The UV absorbance were shown on the left. According to the molecular weight, the four, two, or one ellipse represents tetramer, dimer, or monomer of PKM2, respectively. **E** 32D^BCR-ABL^ cells were ectopically expressed Flag-tagged WT PKM2(PKM2^WT^) or K270R mutant PKM2(PKM2^K270R^). The cell lysate was prepared and analyzed by size-exclusion chromatography. **F** Cytosolic and nuclear extracts were prepared from NB4 cells followed by IP with anti-PKM2 antibody and western blot analysis for the indicated proteins. **G** Immunofluorescent staining of endogenous PKM2 in leukemia cell lines with antibody against PKM2. **H** NIH3T3 cells were co-transfected with PIAS3 and WT or K270 mutant PKM2 plasmids followed by immunofluorescent staining. The right panel showed the amplified images for cells lined with white lines.

SUMOylation may alter multiple aspects of the target proteins including the localization, activity and protein–protein interactions^29,31^. We wondered whether SUMOylation of PKM2 affects its pyruvate kinsase activity. Purified PKM2 were incubated with/without recombinant E1 and E2 followed by measurement of pyruvate kinase activity. As depicted in Fig. 3A, the pyruvate kinase activity of recombinant PKM2 was remarkably inhibited after SUMOlyation reaction. In parallel, size-exclusive chromatography showed a shift of PKM2 tetramer to dimer upon SUMOlyation reaction (Fig. 3B). By contrast, incubation of PKM1 with SUMO E1 and E2 did not affect pyruvate kinase or trigger a conformation change of PKM1 tetramer (Fig. 3C, D). To further verify the relevance of SUMOylation with the conformation switch, WT and K270R mutant PKM2 were ectopically expressed in BCR-ABL-transformed 32D murine hematopoietic progenitor cells(32D^BCR-ABL^) followed by exclusive chromatography. The results showed that WT PKM2 primarily presented in dimeric form while the K270R mutant PKM2 displayed significant amount of tetramer (Fig. 3E). These results demonstrated that SUMOylation dissembled the tetrameric PKM2 to dimer in vitro and in leukemia cells.

Nuclear localization of PKM2 has been documented in multiple circumstances and play a pivotal role in tumor development^7,10,14,19,37,38^. We previously reported nuclear PKM2 modulate DNA damage response through phosphorylating H2AX and interacting with P53^10,11^. Since SUMOylation has been implicated in protein subcellular localization, we next explored the association of SUMOylation and PKM2 nuclear localization. IP assay using antibody against PKM2 after subcellular fractionation showed that SUMOylated PKM2 is dominantly in the nucleus (Fig. 3F). Immunoflurescent staining revealed significant nuclear localization of PKM2 in a number of leukemia cells (Fig. 3G). Previous study suggested that SUMO E3 ligase protein inhibitor of activated STAT3(PIAS3) interacts with PKM2 in U2OS cells^38^. We next co-transfected PIAS3 with PKM2 and observed that PIAS3 induced nuclear localization of WT but not PKM2 with K270R mutation (Fig. 3H). Together, these data demonstrated that SUMO1 modification of PKM2 is associated with nuclear localization.

### SUMOylation of PKM2 block myeloid differentiation

**Fig. 4:**
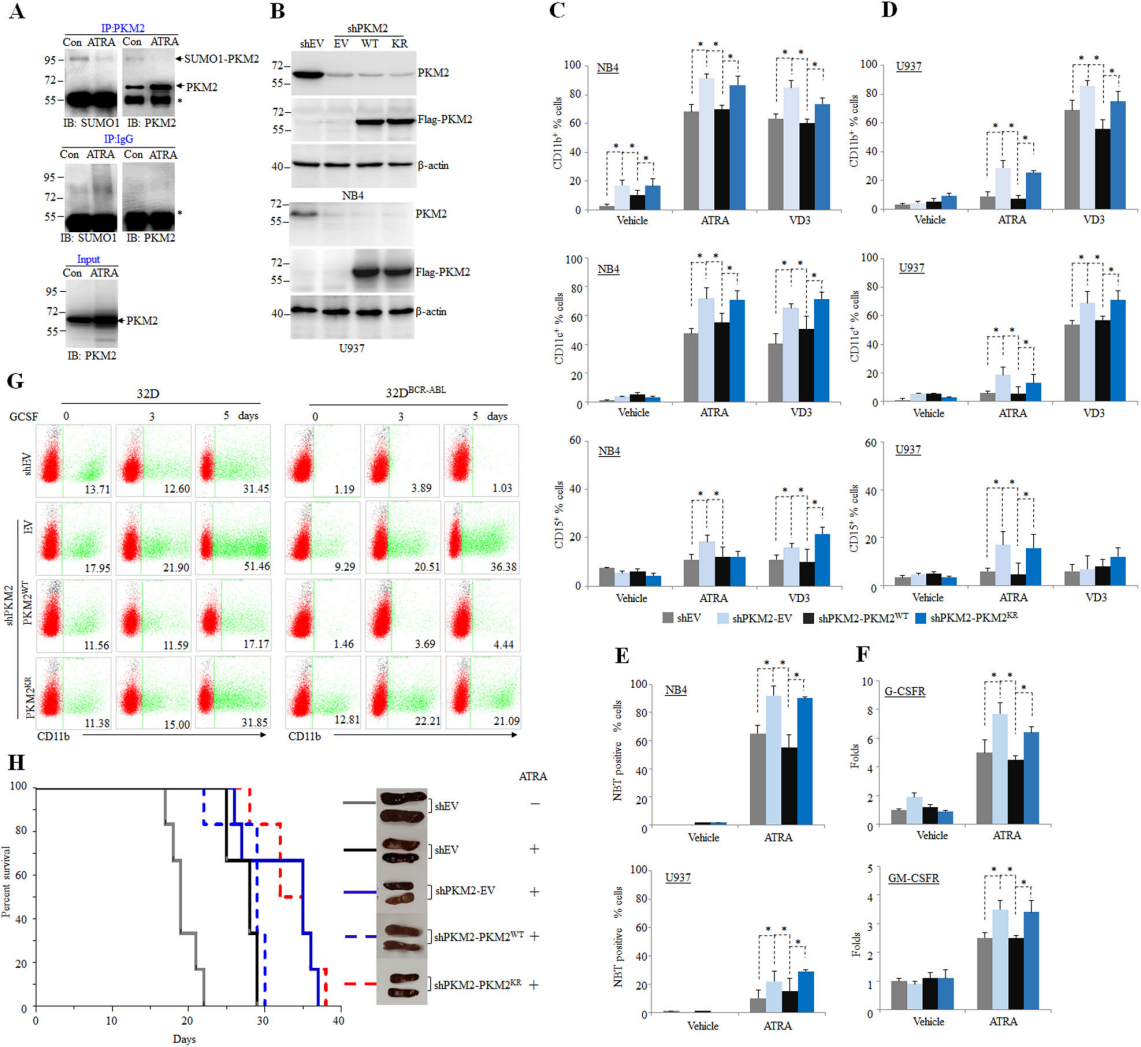
SUMOylation of PKM2 inhibits myeloid differentiation. **A** NB4 cells were treated with 10^−8^ M ATRA for 3 days followed by IP with anti-PKM2 antibody. The SUMO1 modification were analyzed by western blot using anti-PKM2 antibody. **B** Western blot analysis of NB4 and U937 cells in which endogenous PKM2 was stably knocked out and WT PKM2 (WT) or K270R mutant (KR) were re-expressed. **C**–**E** The NB4 and U937 rescue cell lines were treated with 10^−8^ M ATRA or 100 nM VD3 for 3 days and the CD11b-positive, CD11c-positive, or CD15-positive cells were measured by flow cytometry (**C**, **D**) and NBT positive cells were counted (**E**). *p < 0.05 between the line-pointed group. **F** The mRNA level of GCSFR or GMCSFR quantified by real-time RT-PCR were shown. **G** The 32D or 32D^BCR-ABL^ rescue cell lines were treated with 50 ng/ml GCSF for indicated times and the CD11b-positive cells were counted by flow cytometry. Data are shown as the mean ± SEM of three independent experiments. **H** Human NB4 cells (1 × 10^7^) were injected via tail vein into sub-lethally irradiated NSG mice followed by treatment with vehicle or ATRA (10 mg per kg body weight, intraperitoneally) daily for five continuous days a week. The survival data were collected. The representative pictures of spleens from each group were shown on the right.

Myeloid leukemia arises from blockage of the maturation/differentiation of myeloid progenitor cells at different stages with the uncontrolled proliferation and survival advantage of malignant leukemic cells^39,40^. Next, we explored the contribution of PKM2 to differentiation of NB4 and U937 leukemia cells. We treated NB4 leukemia cells with ATRA to induce myeloid differentiation and observed a significant decrease of SUMOylation of PKM2, despite a mild increase of PKM2 protein expression upon ATRA treatment (Fig. 4A). Next, we generated NB4 and U937 cell lines re-constitutively expressing WT or K270R mutant (KR) PKM2 respectively (Fig. 4B), and measured the myeloid differentiation. As evaluated by CD11b, CD11c, and CD15 expression (Fig. 4C, D), NBT reduction (Fig. 4E) and transcription of granulocyte macrophage colony-stimulating factor receptor (GM-CSFR) and granulocyte colony-stimulating factor receptor (G-CSFR) (Fig. 4F), depletion of PKM2 increased ATRA and VD3-induced myeloid differentiation in NB4 and U937 cells, which could be rescued by WT (PKM2^WT^) but not K270R mutant PKM2(PKM2^KR^)^41^. Similar effect was observed in granulocyte colony-stimulating factor (GCSF)-induced myeloid differentiation of murine myeloid progenitor cells 32D (Fig. 4G)^42^. Furthermore, BCR-ABL-transformed 32D cells that were incapable of undergoing myeloid differentiation under GCSF treatment, regained myeloid differentiation upon depletion of PKM2. Re-expression of WT PKM2 but not K270 mutant PKM2 significantly block GCSF-induced myeloid differentiation (Fig. 4G). Subsequently, the impact of PKM2 was further evaluated on xenograft mice. The NB4 rescue cell lines were intravenously injected into sub-lethally irradiated NSG mice, and were treated intraperitoneally with ATRA. Depletion of PKM2 extended survival of leukemic mice undergoing ATRA induction treatment, which was reversed by re-expressing of WT PKM2 but not K270R mutant PKM2 (Fig. 4H). Together, these data demonstrated that SUMOylated PKM2 blocks myeloid differentiation of leukemia cells in vitro and in vivo.

### SUMOylation of PKM2 inhibits RUNX1 expression

As PKM2 catalyzes the final step in glycolysis, we metabolically profiled leukemia cell lines bearing WT or K270R PKM2 by recording the steady-state levels of 13 metabolites central to glucose and energy metabolism through liquid chromatography-MS. We observed that in NB4 and U937 cells, the abundance of pyruvate is decreased upon PKM2 knockout with phosphoenolpyruvate (PEP), the direct substrate of PKM2, accumulated accordingly (Supplemental Fig. 1). By contrast, the metabolites were not altered in the same manner in 32D^BCR-ABL^ cells (Supplemental Fig. 1). The inconsistency between different hematopoietic cell lines indicated that block of differentiation by PKM2 is not attributed, at least not fully, to enzymatic activity.

**Fig. 5:**
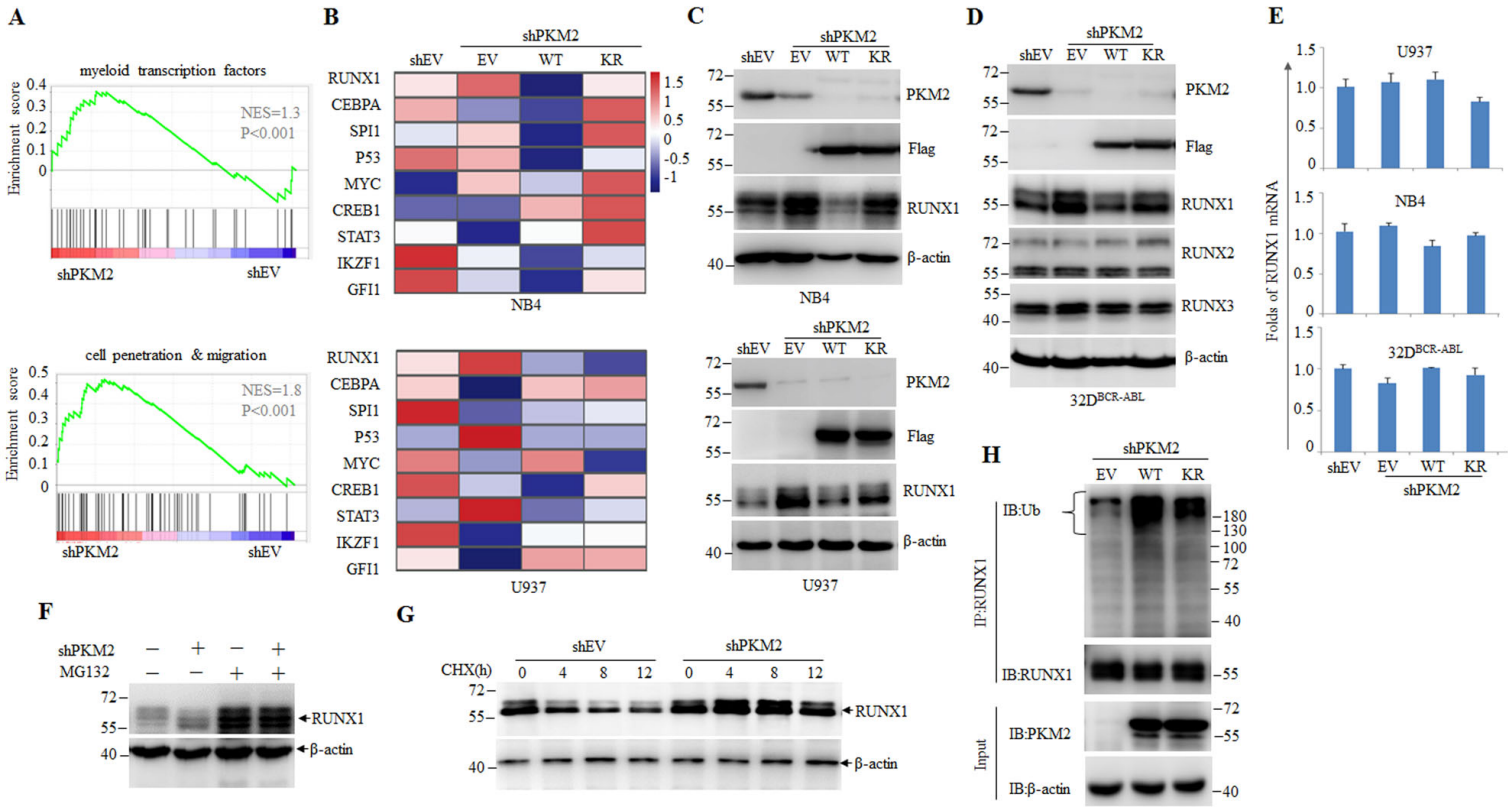
PKM2 controls RUNX1 stability. **A** Gene set enrichment analysis (GSEA) of proteins the in PKM2-depleted U937 and NB4 cells measured by MS. **B** Heatmaps of levels of the indicated transcription factors in NB4 or U937 cells in which endogenous PKM2 was stably knocked out and WT PKM2 (WT) or K270R mutant (KR) were expressed. **C**, **D** NB4, U937, and 32D^BCR-ABL^ rescue cell lines were subjected to western blots and the indicated proteins were detected. **E** The mRNA of RUNX1 was detected by quantitative real-time PCR in NB4, U937, and 32D^BCR-ABL^ rescue cell lines. **F** Western blot analysis of RUNX1 in U937 cells transfected with shRNA targeting PKM2 and treated with MG132 (2 mM for 4 h) as indicated. **G** Western blot analysis of whole cell lysates derived from U937 cells stably expressing shRNA empty vector (EV) or shPKM2 and treated with cycloheximide (CHX, 20 mg/mL) for the indicated time points. β-actin was analyzed as an internal loading control. **H** U937 rescue cell lines were treated with MG132 (2 mM for 4 h) and IP assay were performed using antibody against RUNX1 followed by western blot analysis.

Based on the fact of dominant nuclear localization of PKM2 in hematopoietic and leukemia cells, we next explored the non-metabolic function of nuclear PKM2 during myeloid differentiation. To identify PKM2-dependent molecular events that block myeloid differentiation, we performed unbiased quantitative proteomic analysis on NB4 and U937 leukemia cell lines re-constitutively expressing WT or K270R mutant (KR) PKM2. Gene set enrichment analysis (GSEA) revealed enrichment of myeloid leukemia-associated transcriptional factors and cell penetration/migration in PKM2-depleted cells, indicating a more differentiated state (Fig. 5A and Supplemental Table 1). We next focused on the core transcription factors involved in myeloid differentiation and found that Runt-related transcription factor 1 (RUNX1), a core-binding factor family member, was significant upregulated upon PKM2 depletion, and was inhibited by re-expression of WT PKM2 but not K270R mutant (KR) in both leukemia cells, indicating that RUNX1 is a molecule highly relevant to PKM2 SUMOylation (Fig. 5B and Supplemental Table 2). Western blot further confirmed increased the expression of RUNX1 in leukemia cells lacking PKM2 expression. Re-expression of WT but not KR mutant PKM2 inhibited the expression of RUNX1, but not the other core-binding factor family members RUNX2 or RUNX3 in NB4, U937 and 32D^BCR-ABL^ leukemia cells, suggesting that SUMOylation of PKM2 is essential in modulating RUNX1 (Fig. 5C, D). Notably, RUNX1 mRNA was comparable between leukemia rescue cell lines (Fig. 5E), suggesting that PKM2 decreases RUNX1 protein stability^43^. Indeed, the level of RUNX1 was increased by depletion of PKM2 and this effect was abrogated by the addition of MG132, a proteasome inhibitor (Fig. 5F). The turn-over rate of RUNX1 as measured by cycloheximide (CHX) treatment was also prolonged in the absence of PKM2 (Fig. 5G). Moreover, expression of WT PKM2, but not KR mutant, significantly increased ubiquitination of RUNX1 detected in the presence of proteasome inhibitor MG132 (Fig. 5H).

### SUMOylated PKM2 interacts with RUNX1

**Fig. 6:**
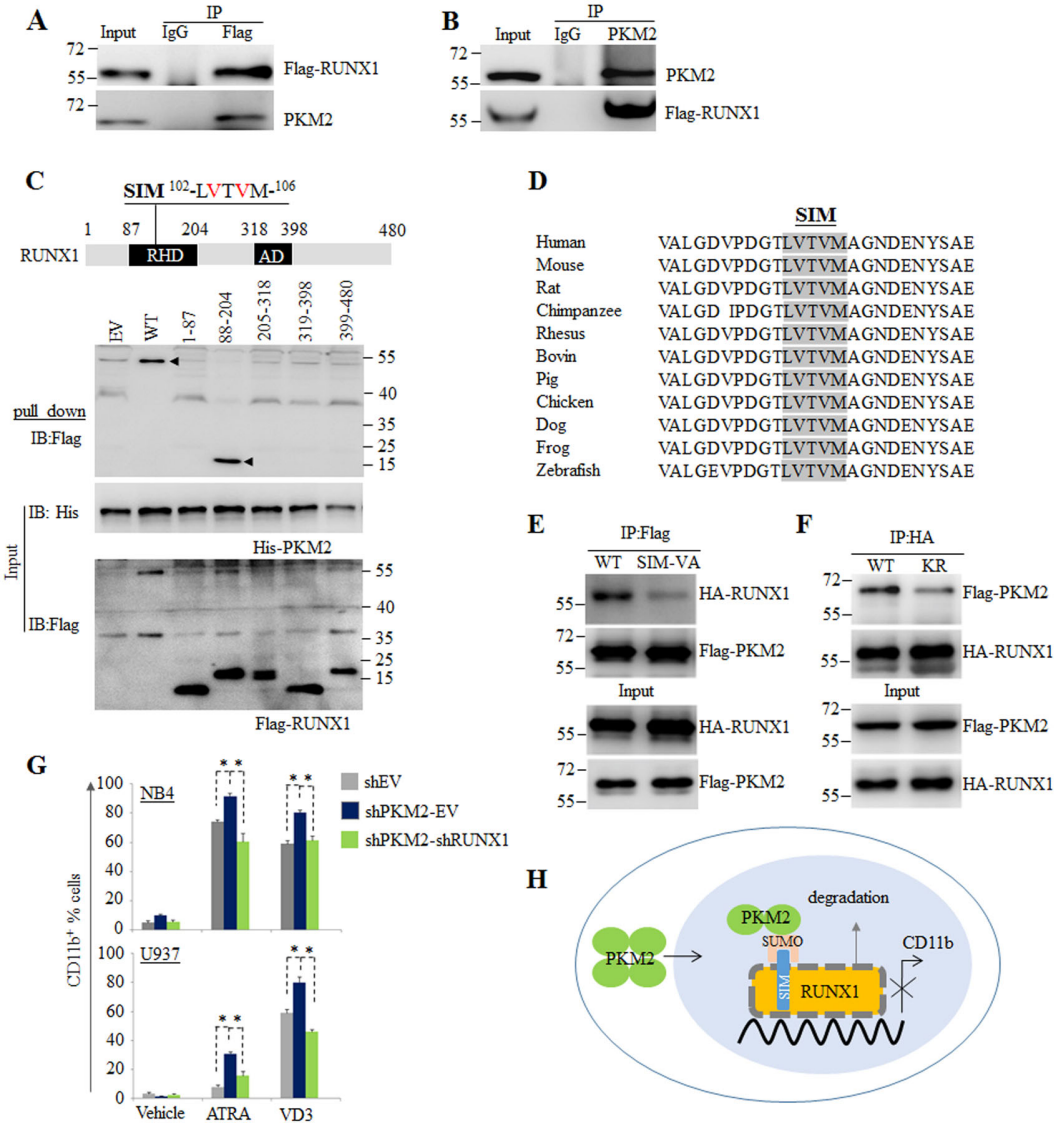
SUMOylation mediates interaction between PKM2 and RUNX1. **A**, **B** IP assay was conducted using antibodies against Flag (**A**) or PKM2 (**B**) in NB4 cells ectopically expressed Flag-tagged RUNX1. **C** IP analysis of interaction between PKM2 and RUNX1 subunits containing the indicated amino acid residues using His-tagged PKM2 and Flag-tagged RUNX1 prepared from NB4 cells. Full-length (WT) or the truncated segments were illustrated by structure schematic diagram. RHD runt-homology domain, AD activation domain, SIM SUMO-interacting motif. The indicated valines (V) in red within the SIM were mutated to alanines (**A**) in the mutants SIM-VA. **D** Sequence alignment of SIM in RUNX1 among different species. **E** WT or SIM-VA mutant RUNX1 were co-expressed with Flag-PKM2 in NB4 cells and IP assay was performed using anti-Flag antibody. **F** WT or K270R mutant PKM2(KR) mutant were co-expressed with HA-RUNX1 in NB4 cells and IP assay was performed using anti-HA antibody. **G** NB4 and U937 cells were infected with CRISPR-Cas9 system targeting PKM2 followed by knocking down RUNX1 with shRNA and treated with ATRA or VD3. The CD11b-positive cells were counted by flow cytometry. *P < 0.05 between the line-pointed group. Data are shown as the mean ± SEM of three independent experiments. **H** SUMOylation of PKM2 dissembles tetrameric PKM2 to dimeric form and induces nuclear translocation where PKM2 promotes degradation of RUNX1, thus blocks the transcription of myeloid associated gene expression.

The fact that PKM2 promotes RUNX1 degradation suggests that the two proteins may physically interact. We next performed co-IP assays in NB4 cells transfected with Flag-tagged RUNX1-expressing plasmids. We showed that endogenous PKM2 protein could be precipitated by anti-Flag antibody (Fig. 6A). Reciprocally, anti-PKM2 antibody could precipitate Flag-RUNX1 protein (Fig. 6B). We further defined the binding domains of PKM2 and RUNX1 using His-tagged PKM2 and Flag-tagged RUNX1 fragments prepared from NB4 cells. As depicted in Fig. 6C, full-length RUNX1 and its runt-homology domain (RHD, aa 87–204) pulled down PKM2 to the similar efficacy, suggesting that PKM2 binds the RHD of RUNX1^44^.

Previous studies have shown that SUMOylated protein can interact with other proteins via SUMO interaction motif (SIM)^45–47^. Sequence analysis of the RHD of RUNX1 identified a “LVTVM” SIM conserved in orthologs from zebrafish to human (Fig. 6D). We next verified the SUMOylation-associated interaction by replacing of the hydrophobic valines to alanines (SIM-VA) in the SIM of RUNX1, and observed strongly reduced binding affinities of PKM2 to RUNX1 (Fig. 6E). On the other hand, SUMOylation deficient mutant PKM2 (KR) had a lower affinity to RUNX1 compared to WT PKM2 (Fig. 6F). These data suggested that SUMOylation of PKM2 is essential for the interaction between PKM2 and RUNX1.

To clarify the role of RUNX1 in PKM2-associated blockage of differentiation, we knocked down RUNX1 in PKM2-depleted NB4 and U937 cells and treated the cells with ATRA and VD3. The results showed that depletion of PKM2 could not promote differentiation in the absence of RUNX1, suggesting an essential role of RUNX1 in mediating PKM2-induced block of myeloid differentiation (Fig. 6G).

## Discussion

The role of PKM2 in tumorigenesis has been controversial. Earlier reports including ours have built up an oncogenic role of PKM2 based on the facts that PKM2, through either metabolic or non-metabolic role, endows tumor cells with growth advantage^10,11^. However, a number of studies also demonstrated that PKM2 is dispensable, or even arrested the progression of cancer^26,48,49^. For example, in vivo growth of established xenograft tumors with HCT116 or RKO colon carcinoma cells is unaffected by PKM2 knockdown^23^. Deletion of PKM2 accelerated tumor formation in a spontaneous breast cancer model^23^.

Instead of focusing on cell proliferation, we herein investigated the potential role of PKM2 in differentiation. We for the first time showed that PKM2 blocked myeloid differentiation in human and murine hematopoietic cells. Intriguingly, this effect of PKM2 required SUMO1 modification at K270, which induced nuclear localization, breakup of tetrameric PKM2 and correspondingly decreased pyruvate kinase activity. Mutation of the K270 abolished the effect of PKM2 on myeloid differentiation, accompanying by more tetrameric form of PKM2. More importantly, endogenous SUMO1 modification and nuclear localization of PKM2 is found to be a common event in hematopoietic and leukemia cells in contrast to solid tumor cells, indicating a pivotal role of PKM2 SUMOylation in leukemogenesis. Notably, the K270 residue of PKM2 is also a key site for phosphoryl transfer from PEP to ADP forming an enolate intermediate and ATP, which determines the enzymatic activity of PKM2^21,50^. Therefore, it is hard to exclude the contribution of pyruvate activity to the effect of PKM2 on cell differentiation. Indeed, we observed a mild decrease of pyruvate and an increase of PEP upon deletion of PKM2 in NB4 and U937 cells yet not in 32D cells. In addition, K270 is 100% conserved among PK isozymes, suggesting that SUMO1 modification might also happen in PKM1 or PKLR which requires further investigations.

RUNX1 is an essential master transcription factor implicated in the differentiation of hematopoietic stem cells as evaluated by the phenotypes of Runx1-deficient mice^51^. As a member of the core-binding factor family, RUNX1 forms a heterodimeric complex via its RHD with core-binding factor β(CBFβ) to exert its sequence-specific transactivation function, while CBFβ is a non-DNA-binding regulatory subunit that allosterically enhances the DNA-binding capacity of RUNX1 and stabilize RUNX1^52^. Since SUMOylated PKM2 also binds with RUNX1 through RHD and downregulates RUNX1 at protein level, we assumed that this binding of PKM2 may impair the interaction between RUNX1 and CBFβ and leads to degradation of RUNX1.

Collectively, we demonstrated a novel role for PKM2 during myeloid differentiation of hematopoietic and leukemia cells. The molecular basis includes the prevalent SUMO1 modification across leukemia cells which breaks up tetrameric PKM2 to dimeric form, and downregulates RUNX1 through direct interaction (Fig. 6H). These results reveal a link between metabolic pathway and leukemia cell differentiation. One may speculate that compounds modulating PKM2 SUMOylation could have potential value for strengthening differentiation therapy for leukemia.

## Materials and methods

### Cells and reagents

Mononuclear cells of bone marrow were collected from acute myeloid leukemia (AML) patients at Department of Hematology of the Second Hospital of Dalian Medical University and were isolated by density gradient centrifugation using Lymphoprep. Patients were diagnosed according to the 2016 revision to the World Health Organization classification of myeloid neoplasms and acute leukemia^53^. Informed consent was obtained from all patients in accordance with the Declaration of Helsinki, and all manipulations were approved by the Medical Science Ethic Committee of Dalian Medical University. Human AML cell lines NB4, U937 and murine myeloid progenitor cells 32Dcl3 (32D) were purchased from Cell Bank of Chinese Academy of Sciences, Shanghai and were maintained in RPMI 1640 medium (Sigma-Aldrich, St Louis, MO), supplemented with 10% heat-inactivated fetal bovine serum (FBS; Gibco BRL, Gaithersburg, MD) in a humidified incubator at 37 °C and 5% CO2/95% air (v/v). Cell line authentication was performed via short tandem repeat profiling. All-trans retinoic acid (ATRA), 1α,25-dihydroxyvitamin D3 (VD3), nitroblue tetrazolium (NBT), MG132 and cycloheximide (CHX) were obtained from Sigma-Aldrich.

### Western blots

Protein extracts were equally loaded on 8–10% SDS-polyacrylamide gel, electrophoresed, and transferred to nitrocellulose membrane (Amersham Bioscience, Pittsburg, PA). The blots were stained with 0.2% Ponceau S red to ensure equal protein loading. After blocking with 5% (w/v) nonfat milk in PBS, the membranes were incubated with indicated antibodies, followed by horseradish perioxidase (HRP)-linked secondary antibodies (Cell Signaling Technology, Danvers, MA). Antibodies against the following proteins were used: PKM2 (Cell Signaling Technology, #4053), PKM1 (Cell Signaling Technology, #7067), HA (Cell Signaling Technology, #7067), Ub (Cell Signaling Technology, #3933), RUNX1 (Cell Signaling Technology, #4334), His (Cell Signaling Technology, #12698), FLAG (Sigma-Aldrich, F1804), SUMO1(Abcam, Cambridge, MA, ab133352) and β-actin (Cell Signaling Technology, #4970). Detection was performed by chemiluminescence phototope-HRP kit (Cell Signaling Technology).

### Vector construction

The plasmids targeting PKM2 were constructed as previously described^10^. Lentiviral vector pLKO.1-shRNA-puro constructs targeting human RUNX1 were constructed as reported^54^. Human RUNX1 cDNA was amplified from U937 cells by RT-PCR and then cloned into pCMV4 vector. Fragments of RUNX1 cDNA were amplified by PCR from pCMV4-RUNX1 plasmids.

### Cell differentiation assay

Cell-surface differentiation antigens CD11b, CD11c, and CD15 were measured using fluorescein isothiocyanate-(FITC) or phycoerythrin (PE)-labeled antibodies with isotype controls (Beckman-Coulter, Krefeld, Germany) via flow cytometry (FACSCalibur, BD Biosciences, San Jose, CA). The antibodies are CD11b (CD11B01), CD11c (11-0116-42) and CD15 (17-0158-42) from eBioscience (San Diego, CA). The NBT reduction was performed as previously described^41^ and 200 cells on each slide were counted under a light microscope (Olympus BX-51; Olympus Optical, Tokyo, Japan) and the percentage of NBT-positive cells was calculated.

### Purification of recombinant proteins

His-tagged WT and mutant PKM2 were expressed in bacteria BL21 (DE3) by induction with 0.5 mM isopropylthiogalactopyranoside (IPTG) at 30 °C and purified from the cytosol of the expressed cells by affinity chromatography on Ni-NTA-agarose (Qiagen, Duesseldorf, Germany).

### Size-exclusion chromatography

Cell lysate was loaded into a Superdex 200 10/300GL(GE Healthcare, Buckinghamshire, UK) column and eluted with elution buffer (50 mM phosphate, 0.15 M NaCl, pH 7.2). Fractions were analyzed by UV absorbance and collected for western blot.

### Immunofluorescence microscopy

Cells were harvested and spotted onto slides coated with poly-l-lysine and fixed with 3.7% (w/v) formaldehyde and permeabilized using a 0.25% (w/v) Triton-PBS solution for 15 min. Cells were blocked with 5% (w/v) BSA-PBS and stained with primary and secondary antibodies. Cells were concurrently stained with 4′,6-diamidino-2-phenylindole(DAPI). Fluorescence signals were detected on a Nikon A1R confocal laser microscope. Secondary antibodies for immunofluorescence staining are Alexa Fluor 488 and Alexa Fluor 555 (Invitrogen Molecular Probes, Carlsbad, CA, A11008 and A31572).

### CRISPR–Cas9 knockout (KO) cells

PKM2-KO cell lines were generated according to a previously described protocol^55^. Briefly, the sequences targeting PKM2 (F: TCTGTGGTTGGACATCTGGC; R: GGCAACACAAGGTGAAGCAG) were cloned into lentiCRISPR v2 vector (Addgene). The sequences targeting EGFP (EGFP-cas9-F: CAC CGGGGCGAGGAGCTGTTCACCG; EGFP-cas9-R: AAACCGGTGAACAGCTCC-TCGCCCC) were used as negative control. The lentiCRISPR v2 vector containing sgRNA, psPAX2, and pMD2G were transfected using Lipofectamine 2000 (Thermo Fisher Scientific) into HEK293T cells. Supernatant were harvested 48 h after transfection and infected the indicated leukemia cells using polybrene (Sigma-Aldrich). The infected cells were selected in medium containing puromycin (Sigma-Aldrich), and single-cell clones were selected and were evaluated by western blot.

### Immunoprecipitation (IP) assay

Whole-cell extracts were prepared in 300 μl of lysis buffer and incubated overnight with indicated antibodies (including anti-PKM2 or anti-Flag) and protein A/G plus-agarose (Santa Cruz Biotechnology, Santa Cruz, CA) at 4 °C. After IP, the beads were intensively washed with washing buffer (50 mM Tris-HCl, pH 7.6; 300 mM NaCl; 1 mM EDTA; 0.5% Nonidet P-40; 10% glycerol). Then the precipitates were analyzed by western blot.

### In vitro SUMOylation reaction-coupled mass spectrometry (MS)

The in vitro SUMOylation reaction were conducted by incubating recombinant PKM2 with purified SUMO E1 (SUMO-activating enzyme subunit 1, SAE1/UBA2), E2 (SUMO-conjugating enzyme 9, UBC9) and SUMO1 proteins. The reaction was ended by adding 2 × SDS lysis buffer. Proteins were dissolved by denaturation buffer (8 M Urea, 0.1 M Tris-HCl, pH 8.5) and centrifuged for 20 min at 14,000 × *g* at 20 °C in YM-10 filter units for three times. Mixed protein lysates were reduced for 1 h at room temperature(RT) by addition of a final concentration of 10 mM dithiothreitol (DTT), and then alkylated for 1 h at RT in the dark by addition of a final concentration of 55 mM iodoacetamide (IAA). The urea solvent of the protein mixtures were exchanged with 50 mM NH_4_HCO_3_ by centrifugation for 20 min at 14 × 000*g* at 20 °C for three times, followed by protein digestion with sequencing grade modified trypsin (Promega, Madison, WI) at a protein-to-enzyme of 50:1 at 37 °C overnight. Tryptic peptides were collected by centrifuge for 20 min at 14 × 000*g* at 20 °C. The tryptic peptides were treated with 1% trifluoroacetic acid (TFA), and were purified using the C18 Ziptips, eluted with 0.1% TFA in 50~70% acetonitrile. The eluted peptides were lyophilized using a SpeedVac (ThermoSavant, Waltham, MA), and resuspended in 10 μl 1% formic acid/5% acetonitrile. All mass spectrometric experiments are performed on a Triple TOF 5600 plus mass spectrometer (AB sciex, Framingham, MA). The peptides mixture were loaded onto a 15 cm with 0.075 mm inner diameter column packed with C18 3-μm Reversed Phase resins, and separated within a 60 min linear gradient from 95% solvent A (0.1% formic acid/2% acetonitrile/98% water) to 38% solvent B (0.1% formic acid/80% acetonitrile) at a flow rate of 300 nl/min. Mass spectrometer settings of typical values were curtain gas = 25, Gas 1 = 3, Gas 2 = 0, an ion spray floating voltage around 2100, and a rolling collision energy voltage was used for CID fragmentation for MS/MS spectra acquisitions. In MS/MS^ALL^ with SWATH (Sequential Windowed Acquisition of all Theoretical fragment ions) acquisition, the Q1 quadrupole is stepped at 25 amu increments covering *m*/*z* 600-1 250, passing a 25 atomic mass unit (amu) window through into the collision cell. The transmitted ions are fragmented and the resulting fragments are analyzed in the TOF MS Analyzer at high resolution. MS raw data was processed by manual identification. Firstly, nineteen fragment ions of SUMO1 side-chain (ELGMEEEDVIEVYQEQTGG) were used as diagnostic ions, and the signals of these nineteen ions were extracted from total ions chromatograph (TIC) data of each window. Secondly, the de-convoluted mass-the precursor ion in the SWATH windows of potential SUMO1-modified peptides matched with the mass of in silico tryptic peptides of FASTA sequence of PKM2 plus tryptic peptide (ELGMEEEDVIEVYQEQTGG) of SUMO1 modification. Finally, the fragment ions with the MS/MS spectrum of SWATH window were mapped with the ions of in silico fragmentation of SUMO1-candidate PKM2 tryptic peptide.

### Multiple-reaction monitoring mass spectrometry (MRM-MS) quantification and data analysis

All MRM experiments were performed on a quadrupole ion trap mass spectrometer (Triple Quad 5 500plus, AB Sciex) coupled to a nano-HPLC system (AB Sciex). The SUMO1-conjugated peptide (ELGMEEEDVIEVYQEQTGG(IIS)KIENHEGVR) of PKM2 identified from in vitro SUMOylation reaction samples were assigned for the MRM (multireaction monitoring) assay. Based on the isotopic standard peptide (EL(C13N15)GMEEEDVIEVYQEQTGG(IIS)KIENHEGV(C13N15)R) MS/MS spectrum, the four isotopic product ions (y′2-757.92(+2), y′3-808.44(+2), y′5-936.99(+2), and b4-862.42(+3)) with the highest intensity for this peptide were selected. Therefore, the counterpart ions for the native SUMO1-conjugated PKM2 peptide (ELGMEEEDVIEVYQEQTGG(IIS)KIENHEGVR) were following four product ions, y′2-754.92(+2), y′3-805.44(+2), y′5-933.99(+2) and b4-860.08(+3). The peptides were eluted to a C18 analytic column (75 μm × 150 mm, 3 μm particle size, 130 Å pore size, at a flowrate of 300 nl/min. The liquid chromatograph was performed using a 60 min gradient comprising 5 min of 5% B (0.1% formic acid/95% acetonitrile), 40 min of 20–50% B, 1 min of 50–85% B, 4 min of 85% B, and a final step of 10 min of 5% B. Each MRM method using a 100 ms dwell time. Based on isotopic MRM analyses, the retention times for those transitions were established. The elution time and shape of all peaks were manually verified. Then the peptides were exported from Skyline. All raw data were analyzed by Skyline to extract and calculate the transition peak areas, and manually inspected by a single experienced person.

### Leukemia Xenograft model

Human NB4 cells (1 × 10^7^) were injected via tail vein into female 8–10-week-old sub-lethally irradiated (2.5 Gy) NOD.Cg-PrkdcscidIl2rgtm1Wjl/SzJ NSG mice (The Jackson Laboratory, *n* = 6 each group). Seven days after injection, the mice were treated with vehicle or ATRA (10 mg per kg body weight, intraperitoneally) daily for five continuous days a week^41,56–59^. The peripheral blood (PB) and bone marrow (BM) cells were collected for flow cytometry analysis. Animal handling was approved by the committee for humane treatment of animals at Shanghai Jiao Tong University School of Medicine.

### Extraction of intracellular metabolites

After rinsed with phosphate buffer saline (PBS), the cells were scraped and lysed in 1 ml H_2_O/methanol (50:50, v:v) solution. Five hundred microliter dichloromethane (DCM) was then added for liquid–liquid extraction. After vortexed for 30 s the mixture was allowed to rest for 20 min at −20 °C and then was centrifuged for 10 min (10,000 × *g*, 4 °C). The upper aqueous content was transferred to clean tubes and was concentrated by Speed-Vac vacuum evaporator (SPD121, Thermo Fisher Scientific, Driesch, Germany) at room temperature. After that, 75 μl ACN/H_2_O (70:30, v:v) solution was added to reconstruct the residue. Then, the supernatant was transferred into auto sampler vials for analysis after centrifugation at 18,000 × *g* for 10 min. Each assay consisted of three biological replicates.

### Targeted analysis on glucose metabolism

The metabolites were detected by using HILIC-UPLC-QTOF-MS. The HILIC-UPLC-QTOF-MS system was equipped with a LC30AD^TM^ UPLC (Shimadzu, Kyoto, Japan) coupled with a TripleTOF^TM^ 5600 plus (AB sciex) QTOF mass spectrometer under negative ion mode. A Waters ACQUITY^®^ UPLC BEH Amide column (1.7 μm, 100 mm × 2.1 mm) was used for chromatographic separation. The column temperature was set at 45 °C, flow rate 0.40 ml/min, sample injection volume 5 μl. The metabolites were separated over a linear gradient from 5% B to 70% B for 10 min (mobile phase A (95% ACN, 0.1% formic acid, and 10 mM ammonium formate) and B ((95% H_2_O, 0.1% formic acid, and 10 mM ammonium formate)). The peak areas of the different metabolites were calculated and determined using Sciex Multiquant software through which the exact mass of the singly charged ion, and retention time was analyzed by the commercially available standard compounds using the same HILIC-UPLC-QTOF-MS system.

### Statistical analysis

The two-tailed student *t*-test for statistical analyses were performed using PRISM 5 (GraphPad, La Jolla, CA). Data were expressed as mean ± SEM. Statistical analyses between the control and treatment groups were performed by standard two-tailed Student’s *t*-test. A value of *p* < 0.05 was considered to be statistically significant. The number of replicates for each experiment was indicated in figure legend. Sample sizes were determined according to investigator’s experience. No sample was excluded from the analyses. Survival differences between groups were calculated with the Kaplan–Meier log rank test in a pairwise manner. Animals were not randomly assigned during collection but according to the strain, sex, and age. The data analysis was single masked. The investigators were not blinded to the group allocation during the experiment.

## Supplementary information

Supplemental text

supplemental table 1

supplemental table 2

supplemental table 3

## Data Availability

The data supporting the finding of this study are available in the supplemental tables and figures. The mass spectrometry proteomics data have been deposited to the ProteomeXchange Consortium (http://proteomecentral.proteomexchange.org) via the iProX partner repository with the dataset identifier PXD023317^60^. Please contact the corresponding author (stove@shsmu.edu.cn or lixia@shsmu.edu.cn) for renewable materials, datasets, and protocols.

## References

[1] Ashizawa K, Willingham MC, Liang CM, Cheng SY (1991). In vivo regulation of monomer-tetramer conversion of pyruvate kinase subtype M2 by glucose is mediated via fructose 1,6-bisphosphate. J. Biol. Chem..

[2] Ikeda Y, Tanaka T, Noguchi T (1997). Conversion of non-allosteric pyruvate kinase isozyme into an allosteric enzyme by a single amino acid substitution. J. Biol. Chem..

[3] Anastasiou D (2012). Pyruvate kinase M2 activators promote tetramer formation and suppress tumorigenesis. Nat. Chem. Biol..

[4] Wong N, Ojo D, Yan J, Tang D (2015). PKM2 contributes to cancer metabolism. Cancer Lett..

[5] Christofk HR (2008). The M2 splice isoform of pyruvate kinase is important for cancer metabolism and tumour growth. Nature.

[6] Yang W (2012). PKM2 phosphorylates histone H3 and promotes gene transcription and tumorigenesis. Cell.

[7] Yang W (2011). Nuclear PKM2 regulates beta-catenin transactivation upon EGFR activation. Nature.

[8] Keller KE, Doctor ZM, Dwyer ZW, Lee YS (2014). SAICAR induces protein kinase activity of PKM2 that is necessary for sustained proliferative signaling of cancer cells. Mol. Cell.

[9] Gao X, Wang H, Yang JJ, Liu X, Liu ZR (2012). Pyruvate kinase M2 regulates gene transcription by acting as a protein kinase. Mol. Cell.

[10] Xia L (2016). A novel role for pyruvate kinase M2 as a corepressor for P53 during the DNA damage response in human tumor cells. J. Biol. Chem..

[11] Xia L (2017). Pyruvate kinase M2 phosphorylates H2AX and promotes genomic instability in human tumor cells. Oncotarget.

[12] Yan JS (2017). The t(8;21) fusion protein RUNX1-ETO downregulates PKM2 in acute myeloid leukemia cells. Leuk. Lymphoma.

[13] Lv L (2011). Acetylation targets the M2 isoform of pyruvate kinase for degradation through chaperone-mediated autophagy and promotes tumor growth. Mol. Cell.

[14] Bhardwaj A, Das S (2016). SIRT6 deacetylates PKM2 to suppress its nuclear localization and oncogenic functions. Proc. Natl Acad. Sci. USA.

[15] Zhou Z (2018). Oncogenic kinase-induced PKM2 tyrosine 105 phosphorylation converts nononcogenic PKM2 to a tumor promoter and induces cancer stem-like cells. Cancer Res..

[16] Hitosugi, T. et al. Tyrosine phosphorylation inhibits PKM2 to promote the Warburg effect and tumor growth. *Sci. Signal.* **2**, ra73 (2009). 10.1126/scisignal.2000431 PMC2812789 19920251

[17] Christofk HR, Vander Heiden MG, Wu N, Asara JM, Cantley LC (2008). Pyruvate kinase M2 is a phosphotyrosine-binding protein. Nature.

[18] Liu F (2017). PKM2 methylation by CARM1 activates aerobic glycolysis to promote tumorigenesis. Nat. Cell Biol..

[19] Wang Y (2017). O-GlcNAcylation destabilizes the active tetrameric PKM2 to promote the Warburg effect. Proc. Natl Acad. Sci. USA.

[20] Anastasiou D (2011). Inhibition of pyruvate kinase M2 by reactive oxygen species contributes to cellular antioxidant responses. Science.

[21] Luo W (2011). Pyruvate kinase M2 is a PHD3-stimulated coactivator for hypoxia-inducible factor 1. Cell.

[22] Hitosugi T (2009). Tyrosine phosphorylation inhibits PKM2 to promote the Warburg effect and tumor growth. Sci. Signal..

[23] Israelsen WJ (2013). PKM2 isoform-specific deletion reveals a differential requirement for pyruvate kinase in tumor cells. Cell.

[24] Hillis AL (2018). PKM2 is not required for pancreatic ductal adenocarcinoma. Cancer Metab..

[25] Lau AN (2017). PKM2 is not required for colon cancer initiated by APC loss. Cancer Metab..

[26] Bluemlein K (2011). No evidence for a shift in pyruvate kinase PKM1 to PKM2 expression during tumorigenesis. Oncotarget.

[27] Geiss-Friedlander R, Melchior F (2007). Concepts in sumoylation: a decade on. Nat. Rev. Mol. Cell Biol..

[28] Dou H, Huang C, Singh M, Carpenter PB, Yeh ET (2010). Regulation of DNA repair through deSUMOylation and SUMOylation of replication protein A complex. Mol. Cell.

[29] Kaur K, Park H, Pandey N, Azuma Y, De Guzman RN (2017). Identification of a new small ubiquitin-like modifier (SUMO)-interacting motif in the E3 ligase PIASy. J. Biol. Chem..

[30] Yeh ET (2009). SUMOylation and De-SUMOylation: wrestling with life’s processes. J. Biol. Chem..

[31] Ritho J, Arold ST, Yeh ET (2015). A critical SUMO1 modification of LKB1 regulates AMPK activity during energy stress. Cell Rep..

[32] Wilkinson KA, Henley JM (2010). Mechanisms, regulation and consequences of protein SUMOylation. Biochem. J..

[33] Eifler K, Vertegaal ACO (2015). SUMOylation-mediated regulation of cell cycle progression and cancer. Trends Biochem. Sci..

[34] Bossis G (2014). The ROS/SUMO axis contributes to the response of acute myeloid leukemia cells to chemotherapeutic drugs. Cell Rep..

[35] Kessler JD (2012). A SUMOylation-dependent transcriptional subprogram is required for Myc-driven tumorigenesis. Science.

[36] Seeler JS, Dejean A (2017). SUMO and the robustness of cancer. Nat. Rev. Cancer.

[37] Yang W (2012). ERK1/2-dependent phosphorylation and nuclear translocation of PKM2 promotes the Warburg effect. Nat. Cell Biol..

[38] Spoden GA (2009). The SUMO-E3 ligase PIAS3 targets pyruvate kinase M2. J. Cell Biochem..

[39] Lu Y (2006). Inducible expression of AML1-ETO fusion protein endows leukemic cells with susceptibility to extrinsic and intrinsic apoptosis. Leukemia.

[40] Lu Y (2008). Multi-sites cleavage of leukemogenic AML1-ETO fusion protein by caspase-3 and its contribution to increased apoptotic sensitivity. Leukemia.

[41] Liu CX (2012). Adenanthin targets peroxiredoxin I and II to induce differentiation of leukemic cells. Nat. Chem. Biol..

[42] Nakajima H, Ihle JN (2001). Granulocyte colony-stimulating factor regulates myeloid differentiation through CCAAT/enhancer-binding protein epsilon. Blood.

[43] Khosravi M (2018). Induction of CD4(+)CD25(+)Foxp3(+) regulatory T cells by mesenchymal stem cells is associated with RUNX complex factors. Immunol. Res..

[44] Sood R, Kamikubo Y, Liu P (2017). Role of RUNX1 in hematological malignancies. Blood.

[45] Zhang Z (2019). OTUB2 promotes cancer metastasis via hippo-independent activation of YAP and TAZ. Mol. Cell.

[46] Meulmeester E, Kunze M, Hsiao HH, Urlaub H, Melchior F (2008). Mechanism and consequences for paralog-specific sumoylation of ubiquitin-specific protease 25. Mol. Cell.

[47] Minty A, Dumont X, Kaghad M, Caput D (2000). Covalent modification of p73alpha by SUMO-1. Two-hybrid screening with p73 identifies novel SUMO-1-interacting proteins and a SUMO-1 interaction motif. J. Biol. Chem..

[48] Cortes-Cros M (2013). M2 isoform of pyruvate kinase is dispensable for tumor maintenance and growth. Proc. Natl Acad. Sci. USA.

[49] Wang YH (2014). Cell-state-specific metabolic dependency in hematopoiesis and leukemogenesis. Cell.

[50] Dombrauckas JD, Santarsiero BD, Mesecar AD (2005). Structural basis for tumor pyruvate kinase M2 allosteric regulation and catalysis. Biochemistry.

[51] Okuda T, van Deursen J, Hiebert SW, Grosveld G, Downing JR (1996). AML1, the target of multiple chromosomal translocations in human leukemia, is essential for normal fetal liver hematopoiesis. Cell.

[52] Blyth K, Cameron ER, Neil JC (2005). The RUNX genes: gain or loss of function in cancer. Nat. Rev. Cancer.

[53] Arber DA (2016). The 2016 revision to the World Health Organization classification of myeloid neoplasms and acute leukemia. Blood.

[54] Goyama S (2013). Transcription factor RUNX1 promotes survival of acute myeloid leukemia cells. J. Clin. Invest.

[55] Ran FA (2013). Genome engineering using the CRISPR-Cas9 system. Nat. Protoc..

[56] Jing Y (2001). Combined effect of all-trans retinoic acid and arsenic trioxide in acute promyelocytic leukemia cells in vitro and in vivo. Blood.

[57] Valiuliene G (2016). Histone modifications patterns in tissues and tumours from acute promyelocytic leukemia xenograft model in response to combined epigenetic therapy. Biomed. Pharmacother..

[58] Lu Y (2019). 2-Bromopalmitate targets retinoic acid receptor alpha and overcomes all-trans retinoic acid resistance of acute promyelocytic leukemia. Haematologica.

[59] Gu ZM (2010). Pharicin B stabilizes retinoic acid receptor-alpha and presents synergistic differentiation induction with ATRA in myeloid leukemic cells. Blood.

[60] Ma J (2019). iProX: an integrated proteome resource. Nucleic Acids Res..

