# Supplementary material for: SUMOylation disassembles the tetrameric pyruvate kinase M2 to block myeloid differentiation of leukemia cells
Source: Cell Death Dis. 2021 Jan 20;12(1):101. doi: 10.1038/s41419-021-03400-9 (PMC7817830; doi:10.1038/s41419-021-03400-9)
Supplement: Supplementary file 1 — Supplemental text [file 41419_2021_3400_MOESM1_ESM.docx]

**SUMOylation disassembles the tetrameric pyruvate kinase M2 to block myeloid differentiation of leukemia cells**

Li Xia^1#^, Yue Jiang^2^, Xue-Hong Zhang^2^, Xin-Ran Wang^1^, Ran Wei^1^, Kang Qin^1^, and Ying Lu^1,2#^

**Supplemental Figure 1** Comparative steady-state metabolomics analyses of subject-matched 32D^BCR-ABL^, NB4 and U937 cells performed via LC–MS. Data are shown as the mean from n=3 experiments. Three independent experiments were performed and data were collected.


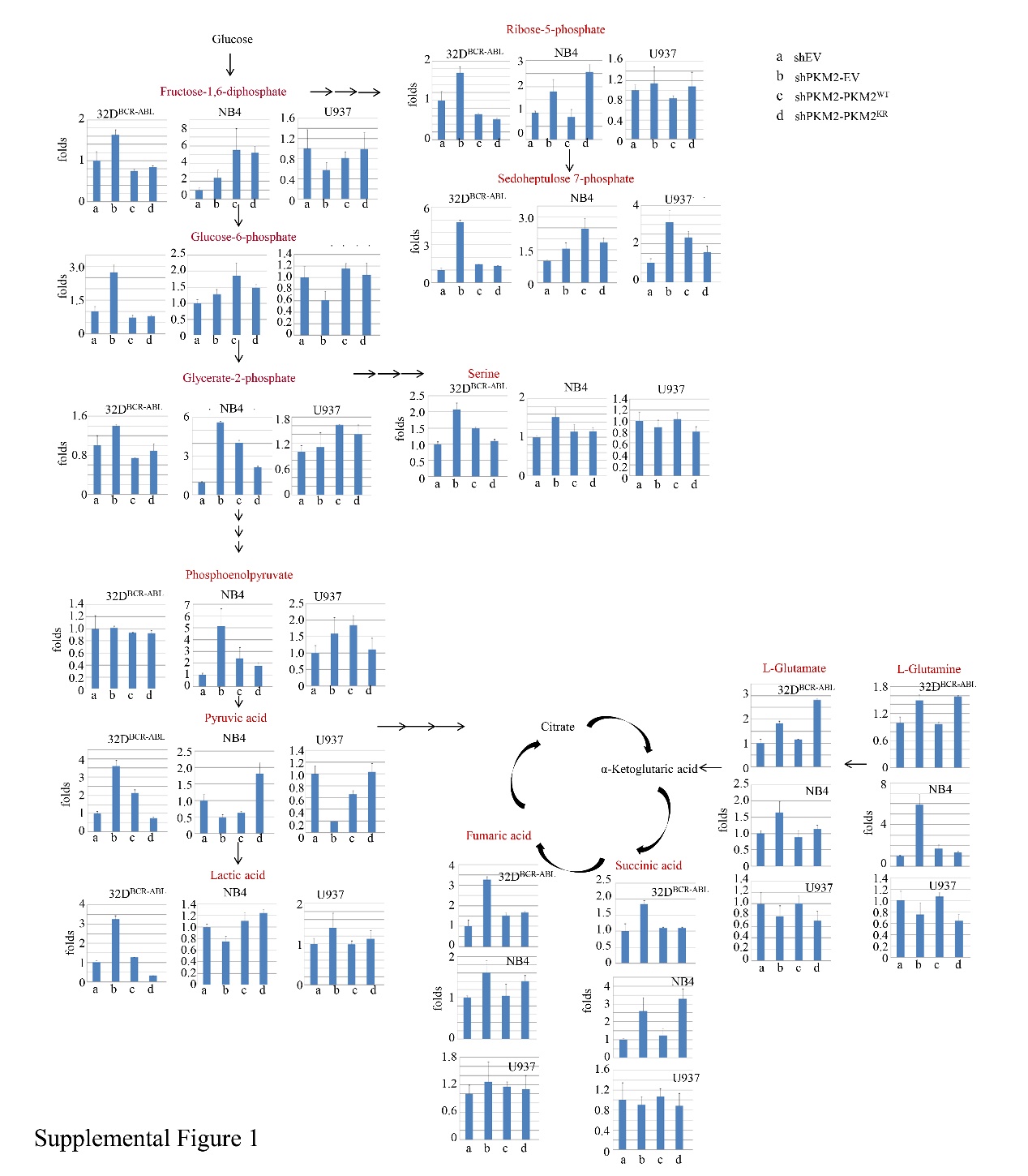


**References**

1. Xia, L.*, et al.* A Novel Role for Pyruvate Kinase M2 as a Corepressor for P53 during the DNA Damage Response in Human Tumor Cells. *J Biol Chem* **291**, 26138-26150 (2016).

2. Goyama, S.*, et al.* Transcription factor RUNX1 promotes survival of acute myeloid leukemia cells. *J Clin Invest* **123**, 3876-3888 (2013).
